# A Case of L-Asparaginase-Induced Severe Hepatic Steatosis With Decreased Serum Cholinesterase Levels

**DOI:** 10.7759/cureus.49787

**Published:** 2023-12-01

**Authors:** Kazuhide Takata, Yuta Nakashima, Satoshi Shakado, Yasushi Takamatsu, Fumihito Hirai

**Affiliations:** 1 Department of Gastroenterology and Medicine, Fukuoka University Faculty of Medicine, Fukuoka, JPN; 2 Department of Internal Medicine, Fukuoka University Faculty of Medicine, Fukuoka, JPN

**Keywords:** vitamin b, l-carnitine, acute lymphoblastic leukemia, serum cholinesterase, hepatic steatosis, l-asparaginase

## Abstract

L-asparaginase (L-Asp) is a useful antileukemic agent for acute lymphoblastic leukemia (ALL); however, it often causes severe liver injury with marked fatty liver. Here, we present a case of L-Asp-induced fatty liver disease in a 21-year-old female patient with ALL. Serum cholinesterase levels, which are usually elevated in fatty liver, decrease at the onset of liver injury. After treatment with L-carnitine and vitamin B complex, the liver injury rapidly improved, resulting in the patient being able to continue subsequent chemotherapy.

## Introduction

L-asparaginase (L-Asp) is a useful antileukemic agent for acute lymphoblastic leukemia (ALL). L-Asp degrades asparagine, leading to asparagine depletion, which in turn prevents the proliferation of ALL cells that do not have asparagine synthase [1]. Severe liver injury owing to L-Asp with marked fatty liver sometimes occurs and leads to a delay or discontinuation of chemotherapy and increased mortality if not diagnosed and treated appropriately [2,3]. Therefore, it is important to appropriately diagnose fatty liver disease at the earliest stage and treat it in patients receiving L-Asp. Herein, we present a case of hepatic steatosis caused by L-Asp.

## Case presentation

A 21-year-old female patient presented to our department for further investigation of elevated liver enzyme levels after the start of chemotherapy. She was diagnosed with ALL a month prior and treated with an ALL-induction chemotherapy. The regimen included cyclophosphamide 1000 mg/m^2^ (day 1), daunorubicin 50 mg/m^2^ (days 1-3), vincristine 1.3 mg/m^2^ (days 1, 8, 15, and 22), L-Asp 6000 units/m^2^ (days 9, 11, 13, 16, 18, and 20), intrathecal (IT) dexamethasone 3.3 mg (day 0), and IT methotrexate 15 mg (day 0). On day 14 of induction, liver enzyme elevation was observed, and liver damage worsened despite the administration of 300 mg of ursodeoxycholic acid; therefore, the patient was referred to our department on day 28. The patient had no history of alcohol intake. Her vital signs were stable, and her physical findings were normal. The patient’s body mass index was 21.4 kg/m^2^. Laboratory tests showed elevated liver enzymes, elevated ammonia from the breakdown of asparagine and glutamine by L-Asp, hypoalbuminemia, and hypocholinesterasemia, all of which were not present at the start of remission induction therapy, in addition to hematological abnormalities due to ALL and chemotherapy (Table 1). Serological markers for hepatitis B, C, Epstein-Barr, and cytomegalovirus were all negative.

**Table 1:** Laboratory data at the time of referral to our department PT, prothrombin time; PT-INR, prothrombin time-international normalized ratio; AST, aspartate aminotransferase; ALT, alanine aminotransferase; GGT, gamma-glutamyl transferase; BUN, blood urea nitrogen; HCV, hepatitis C virus; HBs, hepatitis B surface; HBc, hepatitis B core; EBVCA, Epstein-Barr virus capsid antigen; EBNA, EBV Epstein-Barr virus nuclear antigen; C7-HRP, cytomegalovirus pp65 antigen; ALP, alkaline phosphatase

Laboratory test	Results		Laboratory test	Results	
White blood cell	2500	/μL	IgG	1119	mg/dL
Red blood cell	2.28	10^6^/μL	IgA	95	mg/dL
Hemoglobin	6.9	g/dL	IgM	92	mg/dL
Platelets	59	10^3^/μL	Infectious makers		
Stab cell	3.0	%	HCVAb	(-)	
Segmented cell	70.5	%	HBsAg	(-)	
Lymphocyte	26.0	%	HBsAb	(-)	
Monocyte	0.5	%	HBcAb	(-)	
PT	67	%	EBVCAIgG	40	dil
PT-INR	1.22		EBVCAIgM	<10	dil
Albumin	3.0	g/dL	EBNA	40	dil
Total bilirubin	2.5	mg/dL	C7-HRP	(-)	
AST	293	U/L	HBcAb	(-)	
ALT	229	U/L	EBVCAIgG	40	dil
LD	160	U/L	EBVCAIgM	<10	dil
ALP	92	U/L	EBNA	40	dil
GGT	120	U/L	C7-HRP	(-)	
Cholinesterase	118	U/L			
BUN	19	mg/dL			
Creatinine	0.51	mg/dL			
Ammonia	320	μg/dL			

**Figure 1:**
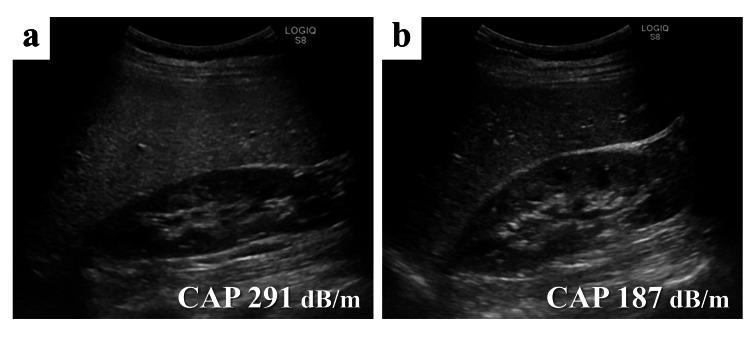
Abdominal images US showing hepato-renal echo contrast on day 28 (a), which disappeared on day 60 (b). CAP, controlled attenuation parameter; US, ultrasonography

Ultrasonography (US) of the liver showed increased liver echogenicity compared to the kidneys, and FibroScan® revealed that the controlled attenuation parameter (CAP) median value was 291 dB/m, suggesting severe hepatic steatosis (Figure 1a) [4]. She was diagnosed with grade 3 L-Asp-induced hepatotoxicity according to the Common Terminology Criteria for Adverse Events (CTCAE) v5 grading criteria.

**Figure 2:**
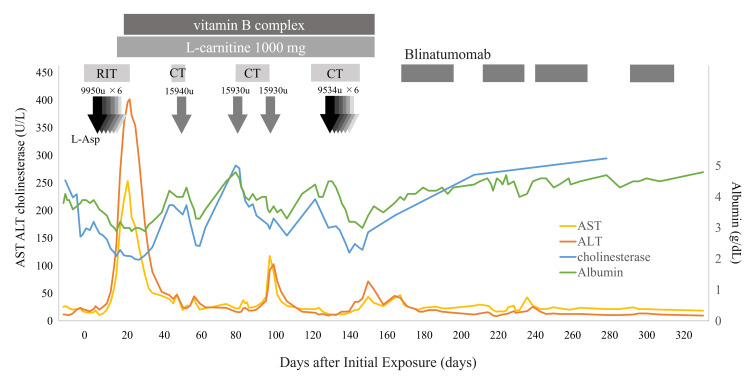
Clinical course RIT, remission induction therapy; CT, consolidation therapy; AST, aspartate aminotransferase; ALT, alanine aminotransferase

The patient immediately received L-carnitine 1000 mg, followed by initiation of a vitamin B complex supplementation (containing 69.16 mg of vitamin B1, 50 mg of vitamin B6, and 500 μg of vitamin B12 per day), which was continued for five months. Subsequently, liver damage exhibited rapid improvement, the fatty liver disappeared, and the median CAP value was normalized to 187 dB/m on US and FibroScan® reexamination one month later (Figure 1b and Figure 2). She underwent consolidation and maintenance therapy, which included L-Asp in her regimen, while continuing L-carnitine and vitamin B complex supplementation (Figure 2). She successfully completed the chemotherapy regimens, although she experienced a flare-up of liver injury, classified as grade 2 according to the CTCAE v5, with L-Asp.

## Discussion

In the present case, the patient had L-Asp-induced liver injury via severe hepatic steatosis. The mechanism underlying L-Asp-induced hepatic steatosis involves asparagine depletion, which hampers protein synthesis and lipoprotein export. Consequently, there is an accelerated accumulation of free fatty acids in the liver owing to impaired mitochondrial β-oxidation and the subsequent development of steatosis. Because L-Asp-induced fatty liver is sometimes fatal, early detection and aggressive therapeutic intervention are recommended [2,3].

One of the hallmarks of this case of ALL with fatty liver is a transient decrease in serum cholinesterase levels. In general, the decrease in serum cholinesterase levels is nonspecific and can occur with nutritional deficiencies and liver dysfunction. Furthermore, it is a known side effect of L-Asp [5]. In our case, these changes were associated with serum albumin levels and were observed after repeated administration of L-Asp (Figure 2). This decrease in serum cholinesterase levels may reflect a decrease in protein synthesis by L-Asp. Meanwhile, serum cholinesterase levels increase, reflecting overnutrition in common fatty liver and nonalcoholic fatty liver disease (NAFLD) [6]. It is clinically important to determine whether fatty liver in ALL patients is newly caused by L-Asp or was present before treatment with L-Asp. However, L-Asp-induced fatty liver disease is more common in patients with obesity, making such a distinction difficult [7]. In cases such as the present case, the decrease in serum cholinesterase levels in fatty liver after L-Asp administration may provide an opportunity to notice differences from NAFLD.

Recently, several reports have shown that carnitine and vitamin B complexes are effective in treating these conditions [8-11]. Carnitine is required for the uptake of fatty acids into mitochondria. Carnitine supplementation promotes β-oxidation and fatty acid breakdown in the liver. Flavin adenine dinucleotide (FAD), which is produced when vitamin B2 is converted, is also required for the β-oxidation of fatty acids. In the present case, after administration of carnitine and vitamin B complex, the L-Asp-induced fatty liver disease quickly improved, allowing repeated re-administration of L-Asp. L-Asp plays a major role in remission induction therapy for ALL, and it is desirable to administer a sufficient dose of L-Asp. Therefore, the treatment of L-Asp-induced hepatotoxicity should be as aggressive as possible to ensure the continuation of treatment.

## Conclusions

L-Asp may result in severe hepatic steatosis due to impaired β-oxidation, and carnitine and vitamin B complexes may effectively ameliorate these conditions. Serum cholinesterase levels may be useful for differentiating L-Asp-induced fatty liver disease from NAFLD.
